# Brusatol Enhances the Chemotherapy Efficacy of Gemcitabine in Pancreatic Cancer via the Nrf2 Signalling Pathway

**DOI:** 10.1155/2018/2360427

**Published:** 2018-04-18

**Authors:** Yukai Xiang, Wen Ye, Chaohao Huang, Dinglai Yu, Hao Chen, Tuo Deng, Fan Zhang, Bin Lou, Jie Zhang, Keqing Shi, Bicheng Chen, Mengtao Zhou

**Affiliations:** ^1^Department of Surgery, The First Affiliated Hospital of Wenzhou Medical University, Wenzhou, Zhejiang Province, China; ^2^Key Laboratory of Diagnosis and Treatment of Severe Hepato-Pancreatic Diseases of Zhejiang Province, Zhejiang Provincial Top Key Discipline in Surgery, Wenzhou, Zhejiang Province, China

## Abstract

Although gemcitabine is the standard chemotherapy treatment for advanced pancreatic cancer, its benefits are quite limited due to prevalent chemoresistance, and the mechanism underlying gemcitabine chemoresistance remains unclear. Currently, Nrf2 has been deemed as a significant contributor to gemcitabine chemoresistance in pancreatic cancer. Brusatol is a unique inhibitor of the Nrf2 pathway, and in previous studies, we determined that brusatol exhibits the effects of growth inhibition and proapoptosis in pancreatic cancer cells. Due to these data, we speculate that brusatol can reverse gemcitabine-induced Nrf2 activation and propose that it can enhance gemcitabine efficacy in treating pancreatic cancer. In this study, we first proved that brusatol can effectively inhibit the Nrf2 signalling pathway and increase ROS accumulation in pancreatic cancer cells. Next, we demonstrated that brusatol can abrogate gemcitabine-induced Nrf2 activation in pancreatic cancer cells. In addition, we discovered that brusatol potentiates gemcitabine-induced growth inhibition and apoptosis in human pancreatic cancer cells. In nude mice with PANC-1 xenografts, treatment with a combination of brusatol and gemcitabine considerably reduced in vivo tumour growth compared with control treatment or treatment with either brusatol or gemcitabine alone. Immunohistochemical staining also showed that Nrf2 expression levels were reduced in brusatol-treated xenograft tumour tissues. In summary, our results suggest that brusatol is capable of enhancing the antitumour effects of gemcitabine in both pancreatic cancer cells and PANC-1 xenografts via suppressing the Nrf2 pathway.

## 1. Introduction

Pancreatic cancer is one of the most fatal diseases that represent the fourth major cause of cancer mortality in western countries with an alarmingly low survival rate in the past 5 years which is less than 5%, even with the best treatments that are currently available [1]. Currently, chemotherapy is widely used for treating this intractable tumour and particularly for treating advanced pancreatic cancer [2]. Gemcitabine (GEM) is the first-line FDA-approved chemotherapy drug for treating pancreatic cancer [2–4], whereas chemotherapy resistance in clinical treatment is difficult to resolve and gemcitabine has limited efficacy in patients [2, 5]. Hence, it is imperative to identify novel therapies for this fatal disease.

NF-E2-related factor 2 (Nrf2) is constitutively active in pancreatic cancer and is correlated with tumour progression and poor prognosis [6–10]. Multiple studies suggest that Nrf2 activation possesses a primary position in the growth, metastasis, and apoptosis of pancreatic cancer [6, 7, 9, 11, 12]. Additionally, recent reports have shown that Nrf2 is important in pancreatic cancer chemoresistance [8, 11, 13]. First, patients with relatively lower expression levels of Nrf2 are more sensitive to chemotherapy [10]. Moreover, gemcitabine can further increase the already high levels of Nrf2 [13]. However, suppressing endogenous Nrf2 can sensitize pancreatic cancer cells to gemcitabine [13, 14]. In addition, the growth of pancreatic cancer xenografts is refrained when applying the combination of the Nrf2 inhibitor and gemcitabine in mice [14]. As reported, in pancreatic cancer cells, gemcitabine can induce reactive oxygen species (ROS); this action serves as an additional anticancer mechanism [13, 15]. However, high expression levels of proteins in the antioxidant defence system, most of which are under the control of Nrf2, can confer chemotherapy resistance [13]. To sum up, results demonstrate that the chemoresistance observed in pancreatic cancer is owing to the Nrf2 pathway. Furthermore, these data suggest that agents that interfere with Nrf2 activation have enormous potential to suppress tumour growth and may also be used to promote the effectiveness of gemcitabine.

Brusatol (BRU) can be isolated from *Brucea*, and it acts as an unparalleled inhibitor of the Nrf2 pathway and enhances the efficacy of chemotherapy in multiple types of cancer cells and A549 xenografts [16, 17]. In our previous study, we found that brusatol exhibits the effects of growth inhibition and proapoptosis in pancreatic cancer cells [18]. Currently, the influence of brusatol on the Nrf2 pathway in pancreatic cancer cells remains unclear, whereas the influence of brusatol on ROS expression in various cancer cells remains controversial. By enhancing ROS production and DNA damage, brusatol can overcome radioresistance in A549 cells [19]. However, when treating HCT116 cells with brusatol under hypoxic conditions, a drastic decrease in intercellular and mitochondrial ROS production was found [20]. Due to these findings, we aimed to determine whether brusatol could suppress the NRF2 signalling pathway in pancreatic cancer cells and to discover its effects on ROS expression. Furthermore, we also investigated whether brusatol could reverse Nrf2 activation caused by gemcitabine and whether brusatol could enhance gemcitabine chemosensitivity either in vitro or in a xenograft mouse model.

Experimental data manifested that brusatol potentiated gemcitabine-induced apoptosis and growth inhibition in pancreatic cancer cells in in vitro experiment and steeply promoted the antitumour effects of gemcitabine on a PANC-1 xenograft pancreatic cancer model. Moreover, our experiments indicated that brusatol could strengthen the anticancer properties of gemcitabine via modulating the Nrf2 signalling pathway.

## 2. Materials and Methods

### 2.1. Materials

Brusatol was purchased from Tauto Biotech (E-1044; Shanghai, China). Gemcitabine HCl for the in vitro experiments was purchased from Shanghai Boyun Biotech Co. Ltd. (BY13174; Shanghai, China), and gemcitabine HCl for the in vivo experiments was purchased from Selleck Chemicals (S1149; Houston, TX, USA). The antibodies used in this study were against Keap1 (AF5266), MDR1 (AF5185), and MRP5 (DF7149) (Affinity Biosciences, OH, USA); Nrf2 (ab62352), NQO1 (ab80588), HO-1 (ab68477), active caspase-3 (ab2302), and Ki-67 (ab16667) (Abcam Inc., MA, USA); and GAPDH (5174S), Bax (2772S), and Bcl-2 (15071S) (Cell Signaling Technology Inc., MA, USA). Brusatol and gemcitabine HCl were dissolved in DMSO and then added to medium to a certain concentration while limiting the DMSO concentration to below 0.1%.

### 2.2. Cell Lines and Cell Culture

The human pancreatic cancer cell lines PANC-1, BXPC-3, and PATU-8988 were obtained from the Chinese Academy of Sciences Cell Bank and maintained in either Dulbecco's modified Eagle's medium (DMEM) or RPMI-1640 medium containing 10% foetal bovine serum (Gibco; Thermo Fisher Scientific, MA, USA). Cells were maintained at 37°C and 5% CO_2_.

### 2.3. Treatment of Cells

The cells were treated in suitable medium with various concentrations of brusatol or gemcitabine alone or their combinations (as indicated in the figure legends), and cells treated with vehicle (PBS) served as a control.

### 2.4. RNA Extraction and Quantitative Real-Time PCR Analysis

Total RNA was isolated with the TRIzol reagent (15596026; Invitrogen, Carlsbad, CA, USA) following the manufacturer's instructions. To quantify the amount of mRNA, cDNA was synthesized from 1 *μ*g of total RNA in a final volume of 20 *μ*L by using the RevertAid First Strand cDNA Synthesis Kit (K1622; Thermo Fisher Scientific, MA, USA). Next, real-time PCR (RT-PCR) was performed with SYBR Green Master Mix (Roche, IN, USA) using the CFX96 Real-Time PCR Detection System (Bio-Rad). *β*-Actin was amplified as an internal standard. All the primer sequences are listed in Table 1.

### 2.5. Western Blotting Analysis

All the proteins were extracted with RIPA buffer (P0013B; Beyotime Biotechnology, Shanghai, China) in combination with phenylmethanesulfonyl fluoride (ST506; Beyotime Biotechnology) and PhosSTOP (Roche). A BCA protein assay kit (P0012; Beyotime Biotechnology) was used to measure the protein concentrations of each individual group. After denaturation, equal amounts of protein were separated by SDS-PAGE and transferred onto PVDF membranes (Millipore, Billerica, MA, USA). After transferring, the membranes were blocked with 5% skim milk for 2 h at room temperature. Then, under the temperature of 4°C, the membranes were probed with primary antibodies overnight. The membranes were washed for three times with Tris-buffered saline containing 0.1% Tween-20 (TBST) and incubated with HRP-conjugated secondary antibodies for one hour at room temperature. At the last stage, the membranes were treated with ECL Western blotting reagents.

### 2.6. Quantification of ROS Levels

Intracellular ROS levels were measured by a Reactive Oxygen Species Assay Kit (E004; Nanjing Jiancheng Bioengineering Institute, Nanjing, China). Briefly, cells were treated with or without brusatol for 24 hours; after that, 10 *μ*M DCFH-DA was added and it was kept for 20–30 min. After, phosphate buffer saline (PBS) was used to wash the cells before trypsinization or direct observation with a Nikon Eclipse TI fluorescence microscope (Nikon Corporation, Tokyo, Japan). Before being resuspended in 500 *μ*L of PBS, cells were collected and washed twice after detachment. To detect fluorescence, a FACSCalibur flow cytometer (BD Biosciences, USA) was used.

### 2.7. Immunofluorescence

Cells were washed with PBS, then fixed in ice-cold paraformaldehyde solution at 4°C for 30 min. Next, they were washed three times by PBS and then permeabilized with 0.5% Triton X-100 at room temperature for 15 min and blocked with 5% bovine serum albumin (BSA) for 30 min. The primary antibody Nrf2 (1 : 200) was incubated at 4°C overnight. Afterwards, the Dylight 488-conjugated donkey anti-rabbit IgG secondary antibody was incubated for 1 h at room temperature in the dark. The incubated cells were washed with PBS, and in order to visualize nuclei, DAPI (C0065; Solarbio, Peking, China) was used. Typical images were captured with an Olympus BX51 fluorescent microscope (Olympus Corporation, Tokyo, Japan). The expression levels of Nrf2 and DAPI were observed separately in the darkness under excitation = 504 nm and emission = 532 nm and excitation = 372 nm and emission = 456 nm.

### 2.8. Cell Viability Detection by Cell Counting Kit 8 Assay

A Cell Counting Kit 8 assay was performed following the manufacturer's instructions. Cell Counting Kit 8 (CCK8; CK04) was purchased from Dojindo (Kumamoto, Japan), and the absorbance of experimental results was detected by an ELISA reader (Tecan, Männedorf, Switzerland).

### 2.9. Colony Formation Assay

An average amount of five hundred cancer cells was seeded into 12-well plates and then treated with 1 *μ*M brusatol or 20 *μ*M gemcitabine alone or in combination for 48 h. After treatment, the cells were permitted to form cell colonies for another 7 days. The cell colonies were fixed in paraformaldehyde solution and stained with crystal violet. Washed and air-dried for 3 times, the stained colonies were able to be photographed by iPhone (Apple, USA).

### 2.10. Apoptosis Assay

Apoptosis analysis was performed following the manufacturer's instructions. The Annexin V-FITC/PI Apoptosis Detection Kit (70-AP101–100; MultiSciences/LiankeBio, Hangzhou, China) was used and analyzed using a FACSCalibur flow cytometer (BD).

### 2.11. Tumour Xenograft Assay

BALB/c homozygous nude mice (male, 4–6 weeks old) were purchased from SLAC Co. Ltd. (Shanghai, China). All animal experiments were following the guidelines set by the Ethical Committee of Wenzhou Medical University and approved by the Laboratory Animal Management Committee of Zhejiang Province. PANC-1 cells (4 × 10^6^) were resuspended in 100 *μ*L PBS and then subcutaneously injected in the right flank of nude mice. Tumour volumes were measured twice a week using vernier calipers and calculated using the following formula: *V* = (*π*/6) × (larger diameter) × (smaller diameter)^2^. One week postinjection, we randomly divided the mice into four groups (*n* = 5/group): (a) control (5 *μ*L of DMSO was dissolved in 200 *μ*L of PBS) was i.p. injected once daily, (b) brusatol (2 mg/kg) was i.p. injected once daily, (c) gemcitabine (100 mg/kg) was i.p. injected twice weekly, and (d) brusatol and gemcitabine had the same schedule as the individual drugs. The mice were closely monitored for 26 days. Then, they were euthanized to weigh the tumours. Each tumour was fixed in 4% paraformaldehyde solution.

### 2.12. Immunohistochemical Staining (IHC)

Tumour tissues were fixed in paraformaldehyde and embedded in paraffin; after that, tumour tissues were excised to 4 mm sections. The slides were stained with antibodies against Nrf2, NQO1, active caspase-3, and Ki-67; then, after washing, these slides were stained with a secondary antibody (PV-6001; ZSGB-BIO, Peking, China) and visualized by the DAB kit (ZLI-9017; ZSGB-BIO). Typical images were able to be captured using an Olympus CKX41SF microscope (Olympus Corporation, Tokyo, Japan).

### 2.13. Statistical Analysis

The data is presented as the mean ± SD. Student's *t*-tests and analysis of variance were used to evaluate the variations among groups. *P* < 0.05 was deemed statistically remarkable.

## 3. Results

### 3.1. Gemcitabine Treatment Induced Nrf2 Activation in Pancreatic Cancer Cells

**Figure 1:**
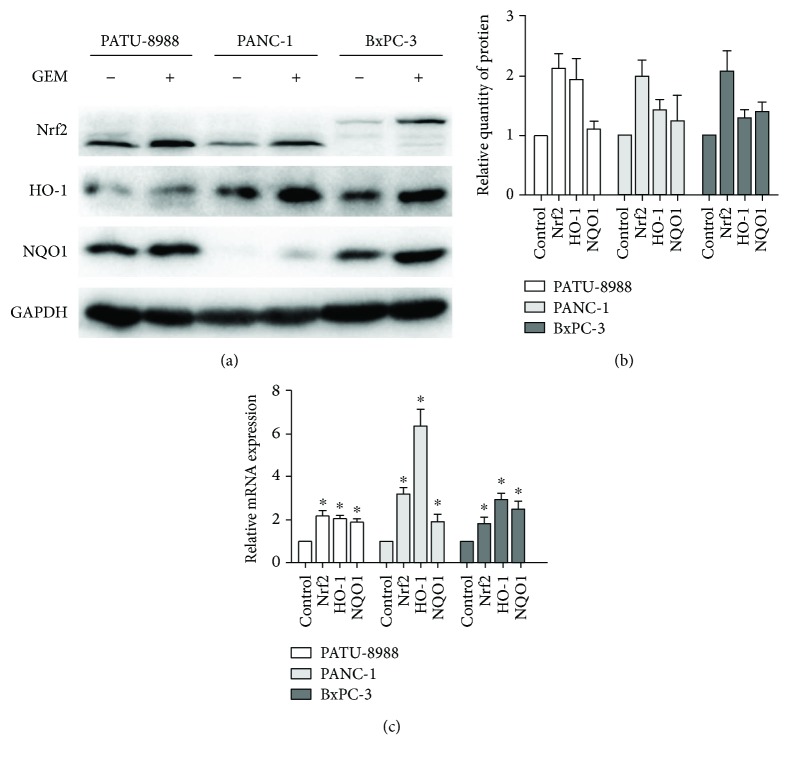
Gemcitabine treatment did induce the activation of Nrf2 of pancreatic cancer cells. (a, b) 5 *μ*M gemcitabine upregulated the protein levels of Nrf2, HO-1, and NQO1 after 48-hour treatments. (c) 5 *μ*M gemcitabine increases the mRNA levels of Nrf2, NQO1, and HO-1 after 48-hour treatments. The data showed representatives of at least three independent experiments. ^∗^*P* < 0.05 compared with the control group.

Aberrant Nrf2 activation is a well-known mechanism of pancreatic cancer resistance to gemcitabine [8, 13]. For the purpose of determining the influence of gemcitabine on the expression of proteins in the Nrf2 pathway, three pancreatic cancer cell lines were exposed to 5 *μ*mol/L gemcitabine for 48 h; then, the protein expression levels were analyzed by Western blotting. Gemcitabine treatment significantly upregulated Nrf2 expression in all three cell lines (Figures 1(a) and 1(b)). The protein levels of the Nrf2 target genes NQO1 and HO-1 were higher in the treated cells than in the control cells. In addition, real-time PCR demonstrated that gemcitabine treatment caused an increase in the mRNA levels of Nrf2, NQO1, and HO-1 (Figure 1(c)). These findings demonstrated that gemcitabine treatment induced Nrf2 activation in pancreatic cancer cells as previously reported [13, 14].

### 3.2. Brusatol Inhibited the Nrf2 Pathway and Increased ROS Accumulation in Pancreatic Cancer Cells

**Figure 2:**
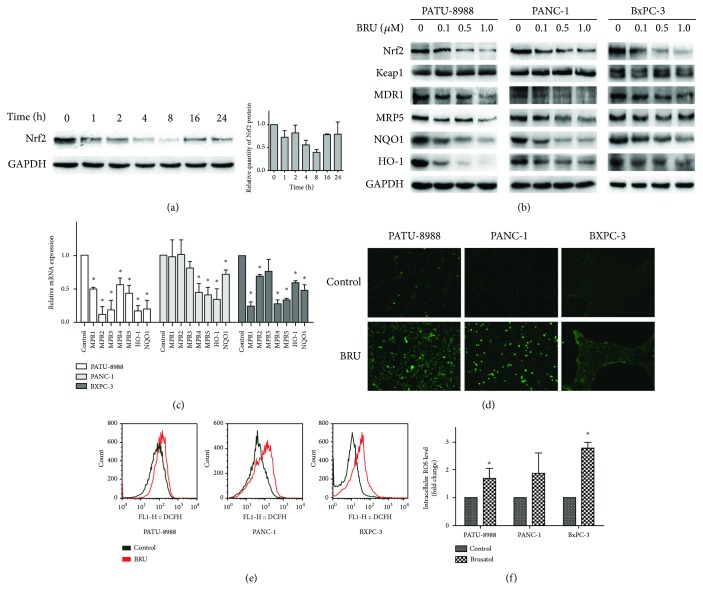
Brusatol inhibited the Nrf2 pathway and increase ROS accumulation in pancreatic cancer cells. (a) 0.5 *μ*M brusatol was utilized to treat the expression of Nrf2 in PATU-8988 cells for the indicated time points. (b) 0.5 *μ*M brusatol lowered the protein levels of Nrf2 and its downstream genes after 8-hour treatments. (c) 0.5 *μ*M brusatol reduced the mRNA level of Nrf2 target genes after 8-hour treatments. (d) Cells were treated with 0.5 *μ*M brusatol for 24 hours, and intracellular ROS generation was analyzed by DCFH-DA using a fluorescence microscope (magnification, 100x). (e) Cells were treated with 0.5 *μ*M brusatol for 24 hours, and intracellular ROS generation was analyzed by DCFH-DA using flow cytometry. (f) The fluorescence intensity of DCF described in (e) was quantitated. The data showed representatives of at least three independent experiments. ^∗^*P* < 0.05 compared with the control group.

In the following, we discovered that compared to control treatment, 0.5 *μ*M brusatol decreased Nrf2 protein levels after just 1 h of treatment. Through the experiment period, the Nrf2 protein levels dropped most remarkably at 8 h, and the effect weakened as time went by. However, this reduction was maintained for up to 24 h in PATU-8988 cells (Figure 2(a)). These data confirmed that brusatol inhibits the Nrf2 pathway. Then, three pancreatic cancer cell lines were exposed to a range of brusatol concentrations for 8 h. Except Nrf2, the protein levels of Nrf2 target genes, containing NQO1, HO-1, MDR1, and MRP5, were reduced, but no effects on Keap1 were observed (Figure 2(b)). Brusatol also decreased the mRNA levels of NQO1, HO-1, MRP1, MRP2, MRP3, MRP4, and MRP5 (Figure 2(c)). In contrast, 0.5 *μ*M brusatol significantly increased ROS accumulation after 24 h of treatment (Figures 2(d)–2(f)). Collectively, the above findings illustrate that brusatol specifically inhibits the Nrf2 pathway and increases ROS accumulation in pancreatic cancer cells.

### 3.3. Brusatol Abrogated Gemcitabine-Induced Nrf2 Activation

**Figure 3:**
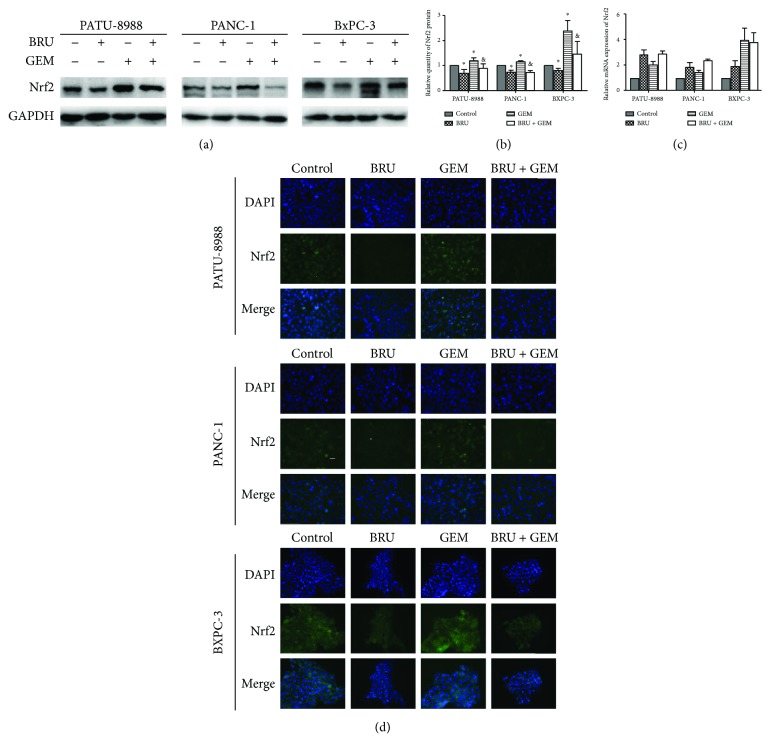
Brusatol could abrogate the Nrf2 activation caused by gemcitabine. (a) The expression of Nrf2 in the indicated pancreatic cell lines that were treated with 0.5 *μ*M brusatol for 8 hours or/and 5 *μ*M gemcitabine for 48 hours was analyzed by Western blot analysis (a, b), real-time PCR analysis (c), and immunofluorescence staining (d). DAPI (blue) was used to indicate the nuclei (magnification, 400x). The data showed representatives of at least three independent experiments. ^∗^*P* < 0.05 compared with the control group. ^&^*P* < 0.05 compared with the gemcitabine group.

Next, we examined the effects of brusatol on gemcitabine-induced Nrf2 activation. First, cells were cultured with 5 *μ*M gemcitabine for 40 h. Next, 0.5 *μ*M brusatol was added, and the cells were incubated for another 8 h. As shown in Figures 3(a) and 3(b), brusatol significantly suppressed gemcitabine-induced Nrf2 overexpression. However, brusatol did not reduce mRNA level of Nrf2 but reversed gemcitabine-induced increases in mRNA levels of Nrf2 (Figure 3(c)). An immunofluorescence assay was then performed to identify the cellular protein levels of Nrf2. We discovered that brusatol treatment lowered the levels of Nrf2 and the nuclear localization of Nrf2 in pancreatic cancer cells (Figure 3(d)). These findings demonstrated that brusatol inhibits gemcitabine-induced Nrf2 activation through reducing the expression of Nrf2 protein in pancreatic cancer cells and that brusatol might intensify the anticancer effects of gemcitabine in pancreatic cancer cells.

### 3.4. Brusatol Enhanced Gemcitabine-Induced Growth Inhibition and Apoptosis in Pancreatic Cancer Cells

**Figure 4:**
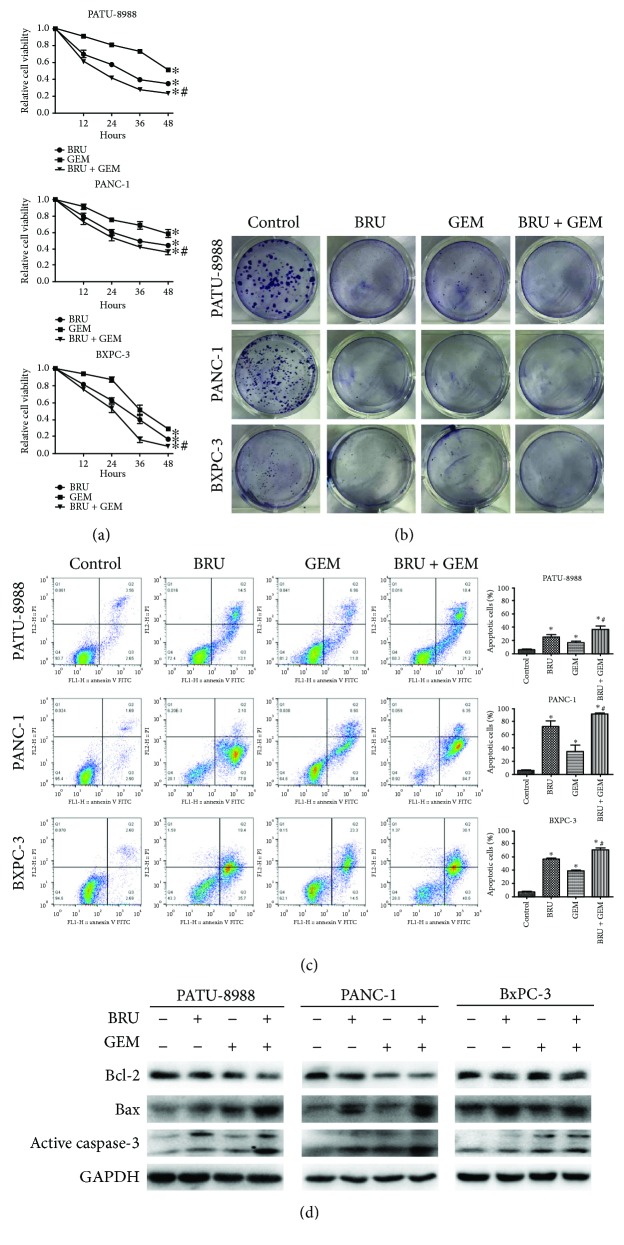
Brusatol enhanced growth inhibition and apoptosis caused by gemcitabine in pancreatic cancer cells. (a) Pancreatic cell lines were treated with 1 *μ*M brusatol or/and 20 *μ*M gemcitabine for the indicated time points. CCK8 assays were used to measure the cell viability. (b) Colony formation assays. Pancreatic cell lines were treated with 1 *μ*M brusatol or/and 20 *μ*M gemcitabine for 48 h. Then, the cells were permitted to form cell colonies for another 7 days. (c) Pancreatic cell lines were treated with 1 *μ*M brusatol or/and 20 *μ*M gemcitabine for 48 h. The cell apoptosis was measured by applying the annexin V-FITC/PI double staining assay. (d) The expression of Bcl-2, Bax, and active caspase-3 in the indicated pancreatic cell lines that were treated with 0.5 *μ*M brusatol for 8 h or/and 5 *μ*M gemcitabine for 48 h was analyzed by Western blot analysis. The data showed representatives of at least three independent experiments. ^∗^*P* < 0.05 compared with the control group. ^#^*P* < 0.05 compared with the brusatol group and gemcitabine group.

To confirm that brusatol potentiates gemcitabine efficacy in pancreatic cancer, we used CCK-8 assays and colony formation assay to investigate the effects of brusatol and gemcitabine separately and in combination on cell viability and growth. For CCK-8 assays, the cells were treated with 1 *μ*M brusatol or 20 *μ*M gemcitabine alone or in combination for 12, 24, 36, and 48 h. Then, we used annexin V/FITC flow cytometric analyses to confirm whether the enhanced cytotoxicity caused by combined treatment with 1 *μ*M brusatol and 20 *μ*M gemcitabine for 48 h was due to apoptosis induction. As expected, we observed that treatment with either brusatol or gemcitabine alone inhibited growth and induced apoptosis in all the three tested cell lines. Compared to treatment with either brusatol or gemcitabine alone, the combined treatment led to obviously further growth inhibition and apoptosis in the cancer cells (Figures 4(a)–4(c)). Furthermore, experiments were carried out to examine the expression levels of apoptosis-related proteins. As shown in Figure 4(d), the levels of Bcl-2, Bax, and active caspase-3 were remarkably varied in the combination treatment group than in the control or gemcitabine-treated group. These results suggested that brusatol enhanced the efficacy of gemcitabine in pancreatic cancer cells.

### 3.5. Brusatol Augmented the Antitumour Activity of Gemcitabine in PANC-1 Xenografts

**Figure 5:**
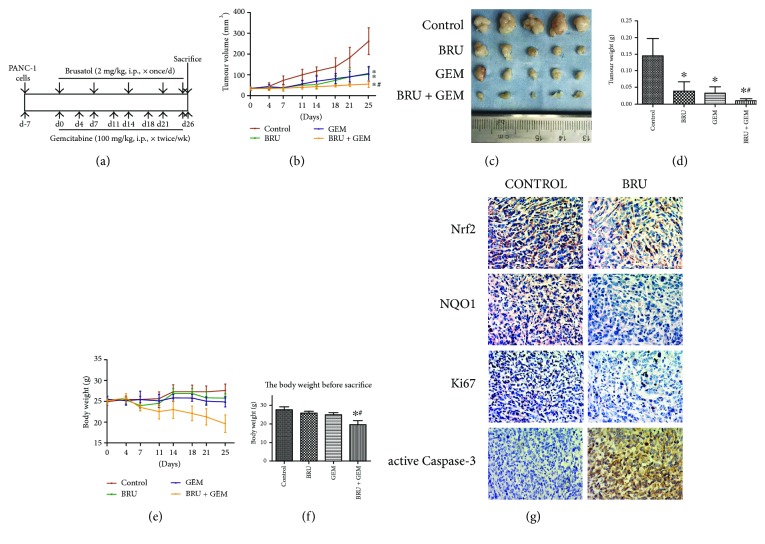
Brusatol augmented the antitumour activity of gemcitabine in PANC-1 xenografts. (a) According to the above flow chart, the experimental design was utilized into in vivo studies. (b) Vernier calipers were used to measure tumour volume, and the following formula was used for calculation: *V* = (*π*/6) × (larger diameter) × (smaller diameter)^2^, at the indicated time points. (c) The excised tumours from mice after the final treatment. (d) The tumour masses were weighed and compared. (e) The body weight was measured twice a week. (f) The body weight before sacrifice was compared. (g) The expression of Nrf2, NQO1, Ki-67, and active caspase-3 in xenograft tumour tissues was observed by immunohistochemical staining (magnification, 400x). ^∗^*P* < 0.05 compared with the control group. ^#^*P* < 0.05 compared with the brusatol group and gemcitabine group.

Building on our in vitro findings, we studied the influences of brusatol and gemcitabine alone or in combination on the growth of human pancreatic tumours subcutaneously implanted in nude mice.  Figure 5(a) is the summary of the treatment schema. Tumours were generated by injecting PANC-1 cells into the subcutaneous tissue of the right flanks of nude mice. A week later, we randomly divided the mice into four groups as stated in Materials and Methods. The treatment was conducted right after randomization continuously for 25 days. The animals were sacrificed on the day after the last treatment. As shown in Figures 5(b)–5(d), tumours treated with either brusatol or gemcitabine alone had significantly lower volumes and weights than the control tumours. Tumours treated with brusatol + gemcitabine were significantly smaller than both the control tumours and the tumours treated with the monotherapies. However, the body weights of the combination-treated nude mice were considerably lower than those of the other groups (Figures 5(e) and 5(f)). These data indicated that the combination treatment exerted an adverse effect on the body weights of nude mice. Furthermore, Nrf2 and NQO1 expression in the xenograft tumour tissues was evaluated by immunohistochemistry. The results proved that both Nrf2 and NQO1 levels were reduced in brusatol-treated xenograft tumour tissues (Figure 5(g)). In addition, IHC analyses of Ki-67 and active caspase-3 indicated tremendously fewer proliferative cells and more apoptotic cells in the brusatol-treated tumours. Together, our results proved the fact that brusatol can augment the antitumour property of gemcitabine by suppressing the tumour expression of Nrf2 in vivo.

## 4. Discussion

Resistance to chemotherapy is one of the most tough clinical challenges concerning with pancreatic cancer treatment. As a standard chemotherapeutic drug, gemcitabine provides limited benefits in treating pancreatic cancer patients [2, 5]. Aberrant Nrf2 activation has been verified as a vital mechanism underlying gemcitabine resistance in pancreatic cancer [8, 11, 13]. Correspondingly, the increase in ROS caused by gemcitabine is considered one of the mechanisms for antipancreatic cancer. However, gemcitabine can further increase the already high levels of Nrf2 which could also enable pancreatic cancer cells to withstand ROS accumulation to cause a chemoresistant pancreatic cancer phenotype; in addition, Nrf2 inactivation is associated with the sensitization of cancer cells to chemotherapy-induced killing in a mechanistic manner [13, 14]. Consistent with recent reports, our observations also show that gemcitabine treatment can induce Nrf2 activation in pancreatic cancer cells. This suggests that agents that interfere with Nrf2 activation may improve the effectiveness of gemcitabine.

Brusatol has been proved to ameliorate chemoresistance in various cancer cells and A549 xenografts by inhibiting the Nrf2 pathway [16, 17]. In our previous study, we found that brusatol not only inhibits growth but also induces apoptosis in pancreatic cancer cells [18]. Here, we have shown that brusatol inhibits the function of Nrf2 and lowers the expression levels of its target genes—members of the multidrug resistance (MDR) family that are involved in drug resistance in pancreatic tumours [21–23]. Consistent with other previous studies [17], the protein levels of Keap1 were not affected by brusatol. Moreover, brusatol is unable to lower the expression of Nrf2 mRNA; we believe that brusatol suppresses the Nrf2 pathway through reduced Nrf2 protein expression independent of Keap1 in pancreatic cancer cells as well as in other cancer cells [16, 17]. Interestingly, in addition to inhibiting the Nrf2 pathway, brusatol increased ROS generation in pancreatic cancer cells which is consistent with the effects of brusatol on 549 cells. It has been reported that brusatol can overcome radioresistance in A549 cells by elevating ROS production and aggravating DNA damage [19]. Our study indicates that brusatol can overcome chemoresistance in pancreatic cancer via inhibiting the function of Nrf2 and increasing the generation of ROS. Thus, we proposed that brusatol can reverse gemcitabine-induced Nrf2 activation. We used Western blotting and immunofluorescence assays to prove this hypothesis. The experiments strongly indicate that brusatol augments the anticancer effects of gemcitabine in pancreatic cancer cells.

As expected, experiments reveal that brusatol enhanced growth inhibition and accelerated apoptosis caused by gemcitabine in vitro. We discovered from previous experiments that brusatol induces apoptosis through the intrinsic pathway [18]. Here, we found that the expression levels of apoptosis-related proteins, such as Bcl-2, Bax, and active caspase-3, vary significantly in the combination treatment group than in the control or gemcitabine-treated group. High expression levels of the antiapoptotic Bcl-2 family have also been implicated in pancreatic cancer chemoresistance [24]. Recently, it has been reported that small interfering RNA oligonucleotides or inhibitors directed against Nrf2 can enhance the sensitivity of pancreatic cancer cell lines to gemcitabine [13, 14]. These experimental results are in line with our observations.

Furthermore, in a subcutaneous pancreatic cancer model, we found results similar to those of our in vitro experiments. Our data clearly showed that brusatol was as effective as gemcitabine at reducing tumour volume, but the combination treatment was apparently more effective at reducing tumour volume than either single-drug treatment. In addition, brusatol weakened proliferation as shown by Ki-67 immunostaining and strengthened apoptosis as shown by increasing active caspase-3 staining in the tumours. In addition, Nrf2 and NOQ1 levels were reduced in brusatol-treated xenograft tumour tissues. These data indicate that the above effects are also related to the downregulation of Nrf2 activity in the tumours. However, the obviously decreased mouse body weights after treatment with the combination of gemcitabine and brusatol are alarming, and there were no notable changes when using either brusatol or gemcitabine alone. It also implied that combined treatment with brusatol and gemcitabine might cause toxicity in human bodies. But several other studies deemed that brusatol exerted no obvious toxicity to the mice [17, 25]; therefore, more experiments are needed to determine the safety of brusatol in the human body.

In conclusion, above-mentioned findings demonstrate that brusatol can inhibit the Nrf2 pathway and increase ROS accumulation. Moreover, brusatol further enhanced the antitumour efficacy of gemcitabine in both pancreatic cancer cells and PANC-1 xenografts partly due to inactivating the Nrf2 pathway. These findings provide new strategies for improving chemotherapy sensitivity in pancreatic cancer.

## Figures and Tables

**Table 1:** Sequences of the primers used for quantitative real-time PCR.

Gene	Forward primer (5′–3′)	Reverse primer (5′–3′)
*β*-Actin	AGAAAATCTGGCACCACACC	AGAGGCGTACAGGGATAGCA
Nrf2	ACCTCCCTGTTGTTGACTT	CACTTTATTCTTACCCCTCCT
NQO1	CATCCCAACTGACAACCA	GAAGCCTGGAAAGATACCC
HO-1	ATTCTCTTGGCTGGCTTC	CTGGATGTGCTTTTCGTT
MRP1	CACGGATAACTGGCAAACCT	ACCCTGTGATCCACCAGAAG
MRP2	TGCTTCCTGGGGATAATCAG	CACGGATAACTGGCAAACCT
MRP3	GGAGGACATTTGGTGGGCTTT	CCCTCTGAGCACTGGAAGTC
MRP4	AAGTGAACAACCTCCAGTTCCAG	GGCTCTCCAGAGCACCATCT
MRP5	AGAACTCGACCGTTGGAATGC	TCATCCAGGATTCTGAGCTGAG
